# Well-Being Factors That Relate to Facebook-Using Older Adults’ Perceived Social Support on Facebook

**DOI:** 10.1093/geroni/igab046.1168

**Published:** 2021-12-17

**Authors:** Amy Schuster, Travis Kadylak, Shelia Cotten

**Affiliations:** 1 Clemson University, Clemson, South Carolina, United States; 2 University of Illinois Urbana Champaign, Champaign, Illinois, United States

## Abstract

The majority of literature on Facebook use and well-being focuses on younger demographics. The number older adults using Facebook continues to increase. Facebook use by older adults has been found to increase well-being and decrease feelings of depression. This study investigates the effect that perceived social support on Facebook may have on loneliness, depression, social support (offline), and fear of missing out (FOMO) for older adult Facebook users. Older adults aged 65 and older in the U.S. completed a Qualtrics survey (N=798). Participants were, on average, 74 years old. Perceived social support on Facebook had a positive association with social support, depression, and FOMO. The results suggest that among Facebook using older adults, higher levels of perceived social support on Facebook were associated with higher levels of social support, feelings of depression, and FOMO. Future research should investigate the possibility that depression could be driving perceived social support on Facebook.

